# Distinct intestinal adaptation for vitamin B12 and bile acid absorption revealed in a new mouse model of massive ileocecal resection

**DOI:** 10.1242/bio.024927

**Published:** 2017-08-17

**Authors:** Yuka Matsumoto, Wakana Mochizuki, Shintaro Akiyama, Taichi Matsumoto, Kengo Nozaki, Mamoru Watanabe, Tetsuya Nakamura

**Affiliations:** 1 Department of Gastroenterology and Hepatology, Tokyo Medical and Dental University, Tokyo 113-8519, Japan; 2 Department of Advanced Therapeutics for GI Diseases, Tokyo Medical and Dental University, Tokyo 113-8519, Japan

**Keywords:** Short bowel syndrome, Ileocecal resection, Intestinal adaptation, Bile acid, Vitamin B12

## Abstract

Ileocecal resection (ICR), one of several types of intestinal resection that results in short bowel syndrome (SBS), causes severe clinical disease in humans. We here describe a mouse model of massive ICR in which 75% of the distal small intestine is removed. We demonstrate that mice underwent 75% ICR show severe clinical signs and high mortality, which may recapitulate severe forms of human SBS, despite an adaptive response throughout the remnant intestine. By using this model, we also investigated whether the epithelium of the remnant intestine shows enhanced expression of factors involved in region-specific functions of the ileum. Cubn mRNA and its protein product, which play an essential role in vitamin B12 absorption in the ileum, are not compensatory up-regulated in any part of the remnant intestine, demonstrating a clear contrast with post-operative up-regulation of genes involved in bile acid absorption. Our study suggests that functional adaptation by phenotypical changes in the intestinal epithelium is not a general feature for nutrient absorption systems that are confined to the ileum. We also propose that the mouse model developed in this study will become a unique system to facilitate studies on SBS with ICR in humans.

## Abstract (precis)

**Summary:** Genes involved in region-specific functions of the distal small intestine show distinct adaptive response following massive ileocecal resection in mice.

## INTRODUCTION

The intestine plays a primary role in the digestion and absorption of nutrients that are essential for life. The small intestine, which consists of the duodenum, jejunum, and ileum, is a long tube-like organ through which our body absorbs most of the nutrients including carbohydrates, proteins, lipids, minerals and vitamins. The large intestine (colon) that begins at the cecum immediately after the ileum is well known as the site of water absorption and also contributes to the absorption of nutrients.

Short bowel syndrome (SBS) is a disorder caused by surgical resection of the small intestine, which leads to a state of malnutrition due to reduced absorptive capacity of the residual bowel (Jeppesen, 2014b). It is well documented that, following resection, the intestine undergoes a process of adaptation, which is manifested by an increase in intestinal caliber, villus height, and crypt depth in the epithelial compartment, all of which serve together to create more mucosal surface area (Tappenden, 2014a; Rubin and Levin, 2016). The severity of SBS is linked to the extent of intestinal resection (Vantini et al., 2004). Patients who undergo massive bowel resection often show severe manifestations that can be life-threatening when the absorptive capacity of the remnant intestine is insufficient to meet the body's needs for nutrients. The clinical manifestation and outcome of SBS also depend on the anatomical site of the bowel resection. It has been shown that ileocecal resection (ICR), which is more common than other types of resection in SBS patients, generally results in more severe disease than jejunal resection (Messing et al., 1999; Tappenden, 2014b). This has been attributed to the lower adaptive abilities of the duodenum and jejunum than the ileum to respond to intestinal resection (Appleton et al., 1987; Carbonnel et al., 1996; Thompson et al., 1999). In addition, the ileum exerts some region-specific functions along the intestinal tract (Tappenden, 2014b), such as absorption of bile acids (BAs) and vitamin B12. Thus, severe manifestations of patients with ICR might also be associated with the lack of potential in the remnant intestine to change their phenotype and compensate for the loss of functions of the ileum.

Mouse models of SBS are invaluable for understanding the pathophysiology of the disease because of their relatively low cost and ease of handling or genetic manipulation. To date, there have been several mouse models of SBS that differ in the extent and site of intestinal resection (Sangild et al., 2014). Among these, Dekaney et al. reported an ICR model in which 50% distal small intestine, cecum, and 1 cm proximal colon were removed (Dekaney et al., 2007). By using this model, they demonstrated that long-term adaptation following intestinal resection involves expansion of epithelial stem cells through the process of crypt fission and intestinal dilatation. Although this model may serve as a useful tool to study mechanisms of intestinal adaptation, it does not mimic severe ICR in humans in that the mice remain in good health only with oral feeding. Regarding other forms of intestinal resection, studies demonstrated that mice that underwent 75% proximal small bowel resection (SBR), in which the distal ileum is left unresected, had worse diarrhea, greater weight loss, and higher mortality than those had 50% proximal SBR (Helmrath et al., 1996; Wakeman et al., 2010). We hypothesized that a mouse model of massive ICR, which shows severe clinical manifestation and outcome in a perceptible manner, would also become a unique system to study the pathophysiology and therapy of patients with massive ICR.

In the present study, we describe a new mouse model of massive ICR in which 75% of the distal small intestine is resected. We demonstrate that mice that underwent 75% ICR show severe clinical signs and high mortality despite an adaptive response throughout the remnant intestine. We also investigated by using this model whether the epithelium of the proximal intestine and colon shows enhanced expression of genes that physiologically act in the ileum to absorb BAs and vitamin B12 as an adaptive response to ICR.

## RESULTS

### Development of a mouse model of 75% ICR

There has been a well-characterized mouse model of ICR in which 50% of the distal small intestine is resected (Dekaney et al., 2007); however, it has never been described whether more extensive ICR leads to more severe form of the disease in mice. We thus attempted to develop a novel mouse model of ICR that allows us to assess the severity of disease caused by the functional loss of the distal small intestine in a perceptible manner by extending the length of intestinal resection.

**Fig. 1.**
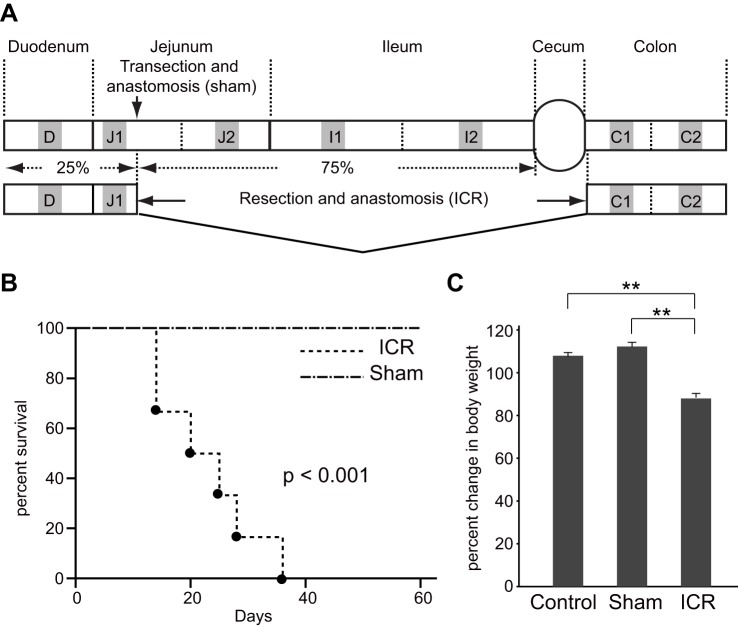
**A new model of 75% ICR mice exhibits severe clinical manifestations.** (A) Schematic representation of the surgeries and intestinal tissue collections. The distal 25 cm of the small intestine (∼75% of the small intestine) and cecum were resected in the 75% ICR model. The site of transection and anastomosis in sham surgery is also shown. The small and large intestines are divided into seven segments in this study and designated as D through C2. The middle 1 cm regions of the segments (gray boxes) were harvested for gene expression and histological analyses. (B) Kaplan–Meier survival curves for mice underwent 75% ICR (*n*=6) or sham operation (*n*=6). Statistical significance was assessed by log-rank test. (C) Mice were randomly assigned to un-operated control (Control), sham operation group (Sham) or those undergoing 75% ICR (ICR). The average body weight on 14 POD in each cohort is shown (*n*=6 for each group). Data are presented as means±s.e.m., **P<0.01 (Mann-Whitney U test).

Our preliminary study showed that the average lengths of the duodenum (from pyloric sphincter to the ligament of Treitz) and the entire small intestine (from pyloric sphincter to the ileocecal junction) in un-operated mice at 12 weeks of age were 3.90±0.12 cm and 33.33±1.12 cm, respectively (*n*=6). Thus, for cohorts of ICR mice, the distal 25 cm of the small intestine was resected with the cecum, and we termed this model 75% ICR (Fig. 1A). For sham-operation cohorts, transection and anastomosis of the bowel were performed 25 cm proximal to the ileocecal junction (Fig. 1A). All mice were maintained on a water or gel form diet for the first 7 days following surgery as described in the Materials and Methods section, and then switched back to a standard diet on post-operative day 8 (8 POD).

First we tracked clinical outcomes of the mice that underwent 75% ICR or sham operation (Fig. 1B). All mice in the ICR group (*n*=6) experienced diarrhea throughout the study period, while the sham operation mice (*n*=6) did not. Although body weight measures of the sham operation mice decreased temporarily in the first week, they returned to their initial levels by 11 POD at the latest (Fig. S1). Among the six mice in the 75% ICR cohort, one showed 30% weight loss (Fig. S1), which was considered as one of the lethal endpoints in our study. In addition, another two mice in the ICR cohort exhibited rectal bleeding at 20 POD and 28 POD, which were also experimental endpoints (Fig. S1). As shown in Fig. 1B, sham operation mice all survived beyond 60 days post-surgery. In contrast, none of the 75% ICR mice survived beyond 35 POD, with a mean survival of 24.5±4.4 days (Fig. 1B). The Kaplan–Meier survival analysis confirms that the mortality rate in the 75% ICR cohort was significantly lower than that of controls (*P*<0.001).

The severity of the clinical manifestations of 75% ICR mice was also evident in other cohorts when assessed by body-weight change on 14 POD. The average weight of the ICR mice (*n*=6) was significantly lower than that of the sham (*n*=6) or un-operated controls (*n*=6) (Fig. 1C). Furthermore, the total cohort of 75% ICR mice showed the overall survival rate of 66.7% (16 out of 24 mice) on 14 POD, which was significantly lower than 94.7% (18 out of 19 mice) in the sham operation group. These findings indicate that the 75% ICR model exhibits severe clinical manifestations that are fatal when maintained only with oral feeding.

### Intestinal adaptation after 75% ICR

**Fig. 2.**
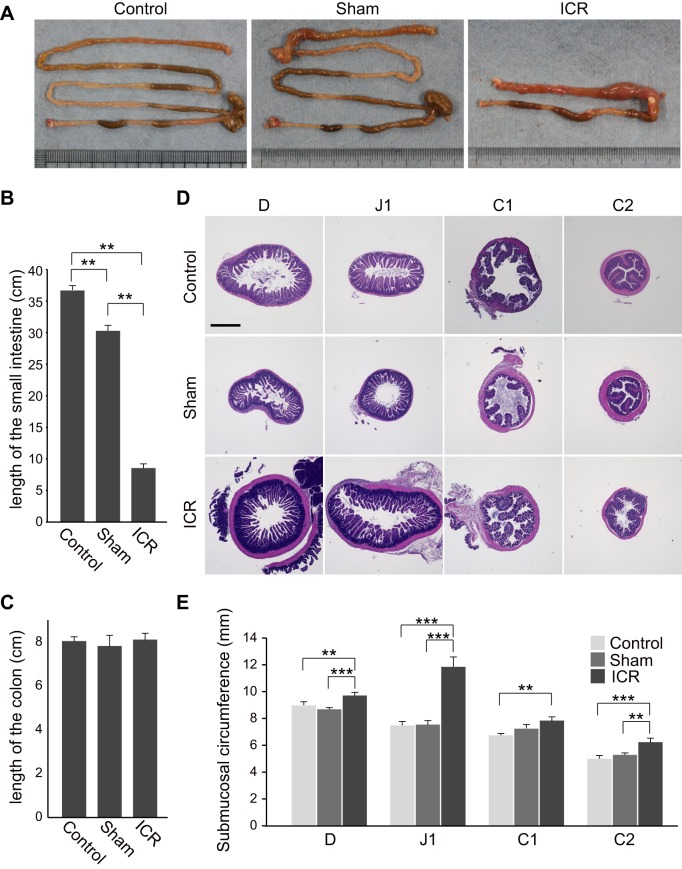
**Gross inspection revealed significant intestinal dilatation in ICR mice.** (A) Photographs of the intestine of mice that were left un-operated (14 weeks of age, left), or underwent sham surgery (middle) or 75% ICR (right) taken at 14 POD. The small and large intestines were placed on the upper side and lower side, respectively. Representative images among those obtained from six mice per group are shown. Immediately after sacrifice on 14 POD, the lengths of the small intestine (B) and the colon (C) of 75% ICR, sham-operated, or age-matched un-operated mice were measured. The average of six mice per group is shown. (D) Micrographs of perpendicular sections of four segments (D, J1, C1, and C2) in the remnant intestine in 75% ICR (ICR) mice and the corresponding segments in un-operated control (Control) and sham operation mice (Sham). Tissues were harvested on 14 POD and representative images are shown. Scale bar: 1 mm. (E) Comparison of intestinal caliber between un-operated control (Control), sham operation mice (Sham) and 75% ICR (ICR) mice on 14 POD. Entire circumference of the submucosa of four intestinal segments (D, J1, C1, and C2) was measured and presented (25 sections, *n*=5). Data are expressed as the means±s.e.m., **P<0.01, ***P<0.001 (Mann-Whitney U test).

To investigate whether adaptive response of the remnant intestine takes place even in this severe disease model, we sacrificed and analyzed 75% ICR mice and sham operation mice on 14 POD. When measured immediately after sacrifice, the total lengths of the small intestine (from pyloric sphincter to ileocecal junction) and the colon (from the distal end of the cecum to the anal verge) of sham-operated mice were 30.17±0.70 cm and 7.70±0.50, respectively (*n*=6) (Fig. 2A, B and C). Those measures of age-matched un-operated mice (14 weeks of age) were 36.50±0.43 cm and 7.92±0.20, respectively (Fig. 2A, B and C). The difference in the small intestine lengths between the un-operated group and sham group was most likely due to the surgical procedures of resection/anastomosis. The lengths of the residual small intestine and the colon of 75% ICR mice were 8.42±0.64 cm and 7.92±0.20, respectively (*n*=6) (Fig. 2A, B and C). Although the difference in the small intestine lengths between the sham and ICR groups was slightly shorter than 25 cm, the length of the resected small intestine, the difference was not statistically significant. These data suggest that, even after massive ICR in adult mice, there may be little, if any, lengthening of residual small and large intestines.

Gross inspection revealed noticeable dilatation of the residual intestine in ICR mice, which was particularly prominent in the region immediately proximal to the anastomosis (Fig. 2A). For detailed histological assessment of the intestine, we discriminated the first and second halves of the jejunum (J), ileum (I), and the colon (C), by tentatively designating these segments as J1, J2, I1, I2, C1, and C2 in this study (Fig. 1A). According to this notation, the residual intestine in the 75% ICR model consisted of the entire duodenum (D), a proximal part of J1, and entire lengths of C1 and C2 (Fig. 1A). We performed histological assessment of residual intestinal segments of ICR mice (D, J1, C1, and C2) and the corresponding intestinal regions of un-operated controls and sham operation mice by examining their perpendicular sections. As predicted, D and J1 segments in ICR mice showed recognizable enlargement of the caliber when compared with those in the other two groups (Fig. 2D). For quantitative assessment, we measured the circumference of submucosa as a measure of mucosal caliber, which allowed us not to take into account variable levels of adaptive thickening of the outer smooth muscle layers (Dekaney et al., 2007). The averages of those circumference measures of all segments (D, J1, C1, and C2) in ICR mice were greater than those in un-operated or sham control mice, although the difference between ICR and sham mice did not reach statistical significance in the C1 segment (Fig. 2E). Among these, the J1 segment of the ICR mice showed the greatest increase of ∼1.5-fold relative to that of the un-operated and sham control (Fig. 2E). This suggests that the increase in bowel caliber occurs differently in response to massive ICR in different segments, with the greatest in the segment immediately proximal to the resected intestine.

**Fig. 3.**
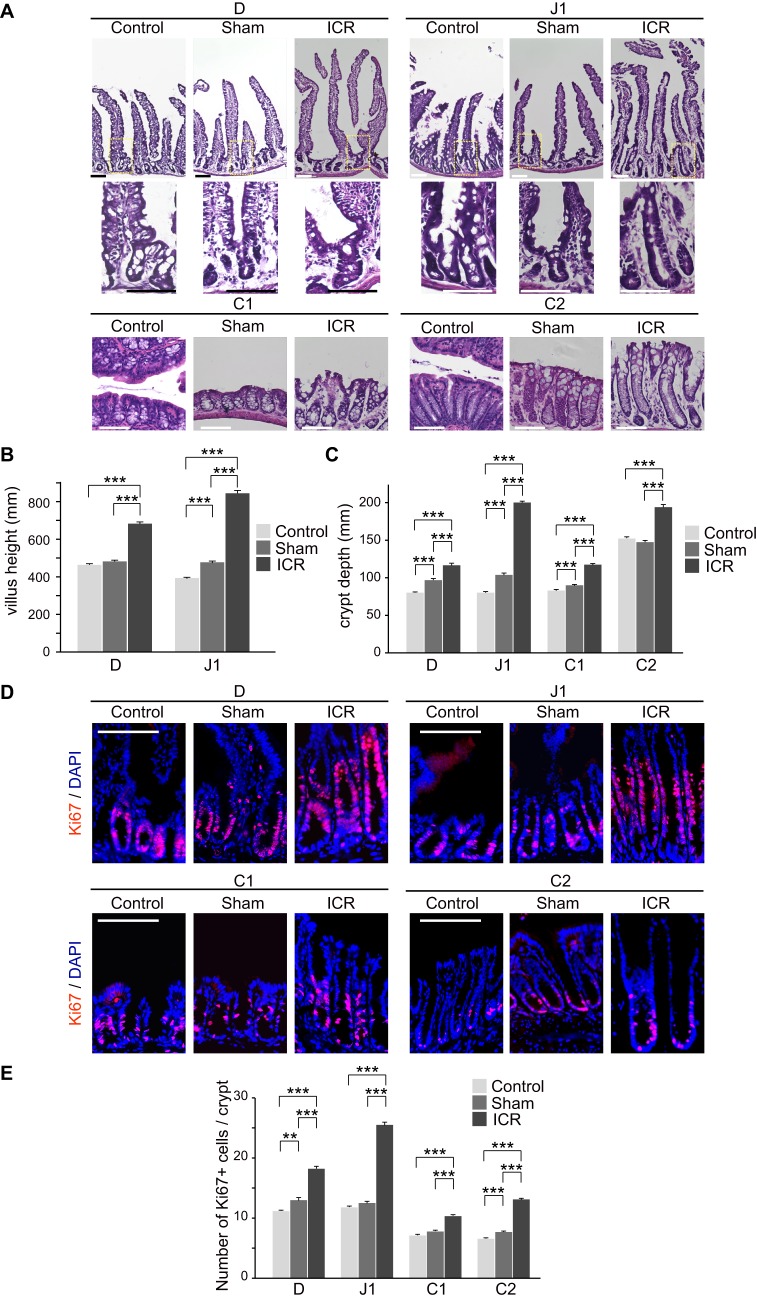
**Histological alteration in remnant intestines in ICR mice on 14** **POD.** (A) Representative micrographs of four segments (D, J1, C1 and C2) in the remnant intestine in 75% ICR (ICR) mice and un-operated (Control) and sham operation mice (Sham). For small intestinal segments (D and J1), views of whole crypt-villus units (top) and magnified views of dotted boxes showing crypts (bottom) are presented. Representative images among those obtained from three mice are shown. Scale bar: 100 µm. (B,C) Measurement of villus height (B) and crypt depth (C) in ICR, sham operation and control mice. Twenty villi and crypts were analyzed per mouse and the data for 100 of those structures are presented (*n*=5). Data are expressed as the means±s.e.m. ***P<0.001 (Mann-Whitney U test). (D,E) Ki67+ cells in the remnant intestine in 75% ICR (ICR) mice and sham control mice (Sham). Representative immunofluorescent images among those obtained from 5 mice are shown in (D). Nuclei were stained with DAPI. Scale bars: 100 µm. (E) Quantification of Ki67+ cells per crypt (D) was performed by analyzing 20 crypts per mouse (*n*=5). Data are expressed as the means±s.e.m., **P<0.01, ***P<0.001 (Mann-Whitney U test).

Further histological examination revealed obvious changes in the epithelial tissues throughout the residual intestine in ICR mice. Villus elongation in the D and J1 segments, and crypt deepening in all segments (D, J1, C1, and C2), were discernible in the ICR mice compared to controls (Fig. 3A). We evaluated these morphological alterations quantitatively by measuring villus height and crypt depth. The ICR mice showed greater villus height as compared with sham operation mice in the D and J1 segments (Fig. 3B). We also observed significant increases in crypt depth in the ICR mice, with profound increase in the J1 region (Fig. 3C). Of note, we found that the villus height and crypt depth of the sham mice were to some extent greater than those of un-operated mice in some intestinal segments. This suggests that there may exist some unknown mechanisms that induce epithelial responses associated with surgical stress or wound healing in anastomoses (Fig. 3B,C). To examine the proliferative activity of epithelial cells, we performed immunohistochemistry for Ki67, a marker protein of proliferating cell populations (Fig. 3D). Consistent with the increase in crypt depth, numbers of Ki67-positive cells were significantly higher in crypts in all segments in ICR mice compared to the sham operation mice (Fig. 3D,E). These results indicate that adaptive changes in the epithelial tissues occurred along the entire length of the remnant intestine even in this lethal model of ICR, involving enhanced proliferation of epithelial cells at the base of crypt structures.

### Adaptive response of BA absorption genes after ICR

**Fig. 4.**
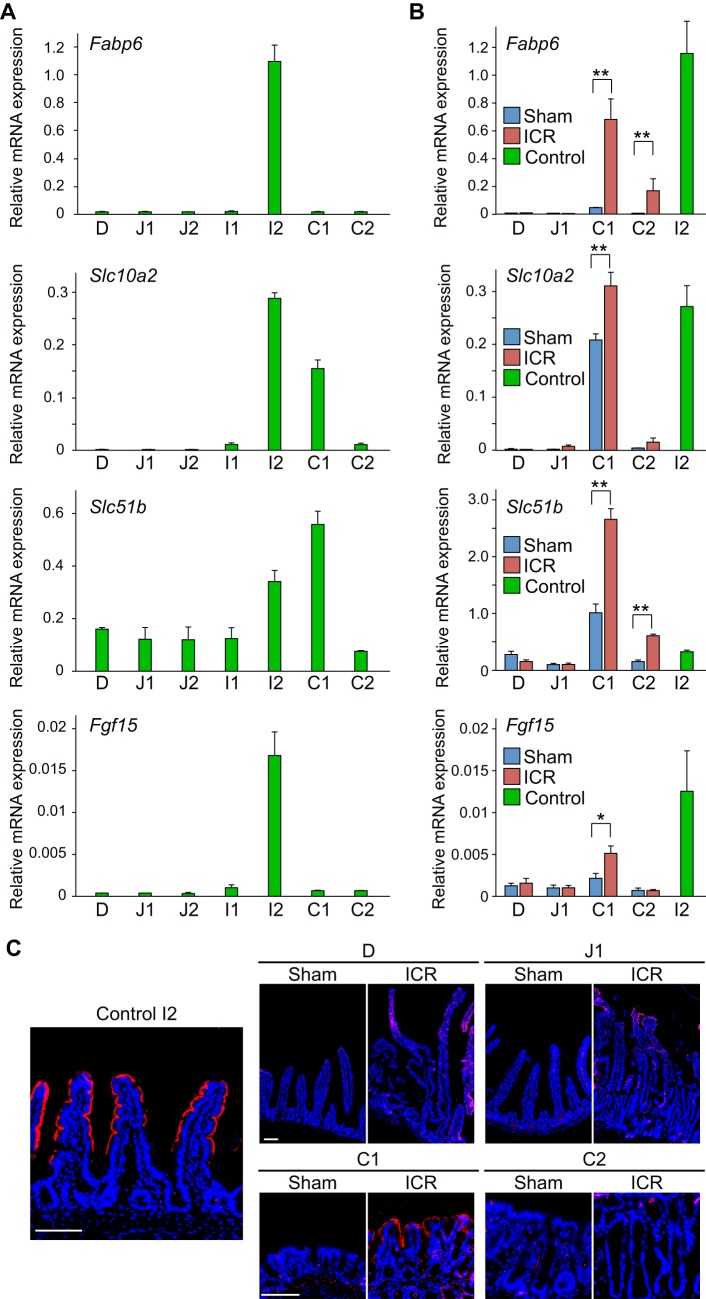
**Expression changes in BA absorption-related factors following ICR.** (A) Expression analysis of genes involved in BA absorption (*Fabp6*, *Slc10a2*, *Slc51b*, and *Fgf15*) along the intestine in un-operated control mice by qRT-PCR. The average value of three samples obtained from three independent mice is shown for each segments. (B) Quantitative gene expression analysis for BA absorption-related genes (*Fabp6*, *Slc10a2*, *Slc51b*, and *Fgf15*) in 75% ICR (ICR) mice and sham control mice (Sham). Total RNAs extracted from four intestinal segments (D, J1, C1 and C2) of ICR and sham control mice on 14 POD, and I2 portion of un-operated control mice were analyzed. Their relative expression levels were determined by double-Δ Ct method using *villin* mRNA as a control. Results obtained from six mice in each group are represented as the means± s.e.m., *P<0.05, **P<0.01 (Mann-Whitney U test). (C) Sections of the I2 intestinal segment of un-operated mice (Control I2), and 4 intestinal segments (D, J1, C1 and C2) of ICR and sham control mice were assayed for protein expression of SLC10A2. Signals yielded by immunofluorescent staining (red) are shown in merged images with DAPI staining (blue). Representative data are shown for samples obtained from three mice in each group. Scale bars: 100 µm.

The ileum exerts some region-specific functions along the intestinal tract. For example, BAs are absorbed mostly at the ileum as a step of their enterohepatic circulation. The genes involved in this process, such as *Fabp6* (Grober et al., 1999), *Slc10a2* (formerly *Asbt*) (Anderle et al., 2005; Middendorp et al., 2014), *Slc51b* (OSTβ) (Middendorp et al., 2014; Wang et al., 2015), and *Fgf15* (Rao et al., 2008) are preferentially expressed in the epithelium of the distal small intestine where their transcriptional regulator, *Nr1h4* (*Fxr*) (Hwang et al., 2002; Makishima et al., 1999), is activated by luminal BAs. A previous study demonstrated that the expression of the BA-related genes is up-regulated in the colonic epithelium in response to massive ICR (Dekaney et al., 2008). However, their adaptive changes in the remnant proximal small intestine remain unclear. We were thus interested in examining expression changes of these BA related genes in the remnant intestine in our massive ICR model in detail. First we performed a detailed assessment of expression patterns of these genes along the entire intestinal tract of un-operated control mice. Isolated intestines were divided into five segments of the small intestine (D, J1, J2, I1, and I2) and two segments of the colon (C1 and C2), and then the extracted RNA from each segment was comparatively assessed by PCR (Fig. 4A). To focus on the gene expression in the epithelium, the relative expression level of each gene was calculated by the conventional double-Δ Ct method by using the *villin* gene as the endogenous control (Wittkopf et al., 2015). It was shown that *Fabp6* mRNA was restrictedly expressed in the I2 segment. *Slc10a2* mRNA was highly expressed in the ileum (I2) and proximal colon (C1) with the highest expression level in I2. *Slc51b* mRNA showed discernible expression throughout the intestine, reaching its highest level in I2 and C1. Expression of *Fgf15* mRNA was confined to the I2 segment. These data indicated that the genes regulating BA absorption are highly expressed in the distal ileum, although their expression patterns along the intestine are individually distinct.

Based on these data, we then examined whether expression levels of BA-related genes changed in the remnant intestine following ICR. We performed qPCR using mRNA isolated from 4 segments (D, J1, C1, and C2) of ICR mice or corresponding regions from sham operation mice at 14 POD. We also simultaneously assessed expression levels of those genes in the I2 segment of un-operated control mice (12 weeks old) for reference. Again, based on the idea that the proportion of epithelial component to the whole tissue would be different between tissues obtained from ICR mice and sham controls, and even among different parts of the intestine, the relative expression level of each gene was calculated by the conventional double-Δ Ct method by using *villin* gene as the endogenous control to see the phenotypic alteration of the epithelial cells in this assay. We found that all genes tested showed higher expression in the C1 segment in ICR mice than in the same segment in sham operation mice (Fig. 4B). *Fabp6* and *Slc51b* also showed significant up-regulation in ICR mice in the C2 segment (Fig. 4B). Regarding the response in the small intestine (D and J1), although *Slc10a2* and *Fgf15* showed higher expression levels in ICR mice than in sham operation mice, they did not reach statistical significance (Fig. 4B). These results demonstrate that the epithelium of the remnant intestine, particularly that of the proximal colon, responds to the loss of the distal small intestine and changes the expression levels of genes involved in BA absorption. We further performed immunohistochemistry for SLC10A2, which showed enhanced expression in the C1 segment following ICR (Fig. 4B). We verified that the anti-SLC10A2 antibody that we used clearly labeled the apical epithelial surface of the upper villi of the I2 intestine obtained from un-operated control mice (Fig. 4C, left). When D, J1, C1 and C2 tissues of sham operation mice were examined under the same experimental condition, they did not yield clear signals (Fig. 4C, right). In contrast, a positive and intense staining was shown on the surface epithelium of the C1 segment of ICR mice (Fig. 4C, right). This indicated that enhanced expression of *Slc10a2* mRNA in the colon (C1) led to increased expression of its protein product in ICR mice.

### Adaptive response for vitamin B12 absorption after ICR

**Fig. 5.**
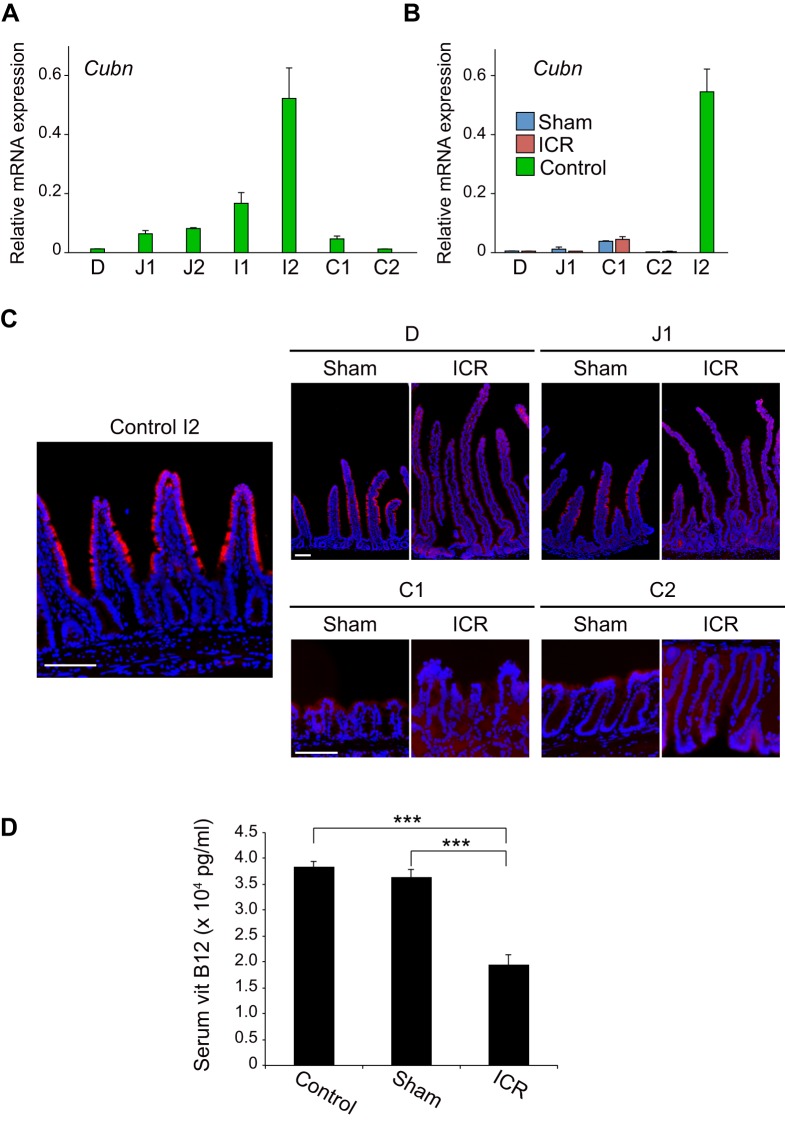
**A lack of functional adaptation for vitamin B12 absorption in ICR mice.** (A) Expression of *Cubn* mRNA along the intestine in un-operated control mice assessed by qPCR. (B) Quantitative analysis of *Cubn* mRNA expression in 75% ICR (ICR) mice and sham control mice (Sham). qPCR was performed as described in Fig. 4B. Relative expression level of *Cubn* mRNA was determined by double-Δ Ct method using *villin* gene as a control. Results obtained from six mice in each of ICR, sham, and un-operated mice (I2 segment) are represented as the means±s.e.m. (C) CUBN protein expression. Sections of the I2 segment of un-operated mice (Control I2), and four segments (D, J1, C1 and C2) of ICR and sham control mice were assayed for CUBN expression. Signals yielded by immunofluorescent staining (red) are shown in merged images with DAPI staining (blue). Representative data are shown for samples obtained from three mice in each group. Scale bars: 100 µm. (D) Serum vitamin B12 in un-operated (Control), sham-operated (Sham) and 75% ICR (ICR) mice on 14 POD. Results are expressed as the means±s.e.m. of six mice for each group. ***P<0.001 (Mann-Whitney U test).

The ileum is also known as the site of vitamin B12 absorption. After binding to intrinsic factor, vitamin B12 is absorbed through a mechanism involving an endocytic receptor, CUBN, which is expressed on epithelial cells of the distal small intestine (Xu and Fyfe, 2000). Insufficient absorption of vitamin B12 following ICR has long been demonstrated by clinical evidence that patients with SBS require parenteral supplementation of vitamin B12 after massive ICR (Allen, 2008; Nightingale et al., 2006). However, it remains unexplored whether the remnant intestine shows any compensatory response to deprivation of the absorption machinery for vitamin B12. To address this, we first analyzed the distribution of *Cubn* mRNA along the un-operated intestine by qPCR (Fig. 5A). In the small intestine, *Cubn* mRNA expression showed an increasing trend from the duodenum toward the ileum, with strong peaks in the ileum (I1 and I2) (Fig. 5A). It then abruptly declined in the colon (C1 and C2), indicating that topographical distribution of *Cubn* mRNA expression is confined to the small intestine under physiological conditions (Fig. 5A). When the expression levels of *Cubn* mRNA in the intestine of ICR mice and those in corresponding intestinal segments of sham operation mice were assessed by qPCR, they did not show significant difference in any segments along the remnant intestine (Fig. 5B). To confirm this observation at the protein level, we conducted immunohistochemistry for the CUBN protein. In the I2 segment of un-operated control mice, CUBN expression was clearly detected on the apical surface of the middle and top parts of villus structures (Fig. 5C, left). In sham operation mice, faint but distinguishable staining was seen on the villi in D and J1 segments (Fig. 5C, right). By contrast, we were unable to detect positive staining in the epithelium of any segment of the intestine in ICR mice (Fig. 5C, right). These data demonstrate that, unlike the genes related to BA absorption, *Cubn* mRNA and its protein product are not compensatory up-regulated in the intestine in response to ICR. We further assessed whether the lack of compensatory expression of CUBN in the remnant intestine might indeed lead to malabsorption of vitamin B12 in this ICR model. When analyzed on 14 POD, serum levels of vitamin B12 of sham operation mice were comparable to those of un-operated mice fed with a standard diet (Fig. 5D). By contrast, serum vitamin B12 of ICR mice was significantly lower than that of sham operation mice (Fig. 5D). These collective observations showed that, although structural and histological changes are induced as an adaptive response in 75% of ICR mice, the malabsorptive state of vitamin B12 and/or the resultant alteration of its luminal concentration did not drive the remnant intestine to change its phenotypes to compensate for the loss of its absorption machinery.

## DISCUSSION

In the present study, we developed and characterized a mouse model of massive ICR in which 75% of the distal small intestine is resected. The mice that underwent 75% ICR showed severe clinical signs and resulted in 100% lethality within 35 days post surgery. Histological assessment showed that this massive ICR model induces intestinal adaptation, which is characterized by increases in villus height, crypt depth, bowel caliber and cellular proliferation. These histological features are distinct from those observed in mouse models of intestinal inflammation, which typically include villous blunting or atrophy in the small intestine (Erben et al., 2014). In addition, we found that the number of colonic goblet cells does not decrease but rather increases in 75% ICR mice (data not shown), which also distinguishes this massive ICR model from inflammatory intestinal disease models in mice (Erben et al., 2014). Thus, although the cause of death of these animals remains undetermined, severe clinical manifestations such as persistent diarrhea and the significant loss of body weight are likely to be associated not with inflammatory responses post-surgery but with malabsorption and the resultant severe malnutrition in this model. Regarding the model of proximal intestinal resection, studies have shown that extended resection of the bowel is associated with increased mortality (Helmrath et al., 1996; Wakeman et al., 2010). Wakeman et al. further showed that resection greater than 50% of the proximal small intestine in mice results in greater mortality without further magnification of the adaptive response (Wakeman et al., 2010). This suggests that performing resections greater than 50% is not necessarily meaningful to study the mechanisms underlying adaptation. Nevertheless, we still consider characterization of the extreme model of ICR to be worthwhile in order to facilitate studies on SBS. For severe cases of SBS due to extensive small bowel resection, long-term parenteral nutrition (PN) or intestinal transplantation should be considered as the therapeutic options. However, because of the risk of severe complications of long-term PN and the need for life-long immunosuppression following intestinal transplantation, the development of novel treatment strategies for severe SBS patients is still an intense focus of research (Buchman et al., 2003). Factors that regulate intestinal adaptation in the remnant intestine have been intensively investigated as potential targets to improve intestinal absorptive capacity in SBS patients (Jeppesen, 2014a; Neelis et al., 2016; Rubin and Levin, 2016; Tappenden, 2014a). Several tissue-engineering approaches have also been taken in attempt to increase functional mucosal surface area of the small intestine (Choi et al., 1998; Finkbeiner et al., 2015; Grant et al., 2015; Walker et al., 2006). One of the advantages of mouse studies on SBS would be the ability to screen and examine such potential therapies with relatively low cost and ease of handling. In this regard, we believe that the 75% ICR mouse model characterized in this study, together with previously described other forms of massive SBR in mice (Helmrath et al., 1996; Wakeman et al., 2010), may serve as a unique system to test whether a particular therapy could improve the clinical outcome of SBS with massive SBR, which does not allow patients to survive on enteral feeding alone because of severe manifestations.

By employing the 75% ICR mouse model, we also assessed whether the epithelium of the remnant intestine shows enhanced expression of genes preferentially expressed in the ileum as an adaptive response. By using different animal models of SBS, several studies have investigated whether restoration of specific functions of the intestine occurs in the remnant intestine (Gillard et al., 2016; Iqbal et al., 2008; Musch et al., 2002). Irrespective of whether or not the restoration is fully sufficient to compensate for loss of function, such a phenomenon is referred to as ‘functional’ adaptation and reported to be mediated by two mechanisms. One is the adaptive response associated with an increase in cells relevant to the function of interest, but not with cellular phenotypical changes (Gillard et al., 2016; Iqbal et al., 2008). The other is the adaptation characterized by changes in cellular functions that occur in addition to overall structural adaptation, which was exemplified by the increased expression of genes encoding apical membrane Na/H exchangers, *Slc9a2* (*Nhe2*) and *Slc9a3* (*Nhe3*), following SBR (Musch et al., 2002). In the present study, we focused on the cellular changes following ICR, and thus investigated expression changes of the genes involved in BA and vitamin B12 absorption by using an epithelial marker *villin1* as a reference gene.

We showed that the genes involved in BA absorption that we assessed were all up-regulated in the proximal colon (C1) in 75% ICR mice. These results reconfirmed the data demonstrated in an earlier study where a 50% ICR mouse model was used to analyze expression of the same set of genes in the proximal colon (Dekaney et al., 2008). A number of mechanisms have been proposed for intestinal adaptation (Rubin and Levin, 2016; Tappenden, 2006, 2014a), and factors that directly or indirectly act on the intestinal mucosa may influence those mechanisms. In the remnant intestine that is distal to the anastomosis, increased loads of nutrients may have direct effects on mucosal adaptation. Other factors, such as altered hepatobiliary secretions and the release of humoral factors, may indirectly act on both the proximal and distal intestine to the anastomosis. Dekaney et al. postulated that the increased BA load to the colonic lumen might be the major mechanism that stimulates NR1H4-dependent transcription and enhances expression of genes such as *Fabp6* and *Fgf15* in colonocytes after ICR (Dekaney et al., 2008). Our study further demonstrated that some of those BA-related genes (*Fabp6* and *Slc51b*) were induced even in the distal colon (C2). Therefore, we also favor the idea that direct exposure of the remaining colon to high concentrations of luminal BA is likely to be the major mechanism by which genes needed for BA absorption are up-regulated.

By contrast, we presented that compensatory up-regulation is not induced for *Cubn* mRNA and its protein product in any segments after ICR. This suggests that neither direct nor indirect factors influence the regulatory mechanism of *Cubn* expression in this ICR model. Regulatory mechanisms of *Cubn* expression in the physiological intestine remain unclear. A previous study demonstrated that the *Cubn* gene undergoes monoallelic silencing in the small intestine, and peroxisome proliferator-activated receptor α (PPARα) and γ (PPARγ) transcription factors play important roles in its gene transcription on its active allele (Aseem et al., 2013). However, considering that PPARα (de Vogel-van den Bosch et al., 2008) and PPARγ (Bouguen et al., 2015) are broadly expressed in the small intestine and colon, respectively, it is unlikely that preferential expression of *Cubn* in the distal small intestine depends solely on its transcriptional regulation by PPARs. It may thus be of importance to explore the mechanism of regionally regulated *Cubn* expression to understand the mechanisms for the lack of its functional adaptation in the remnant intestine following ICR. Our data also demonstrate that functional adaptation to compensate for the loss of distal small intestine is not a general feature for nutrient absorption systems in the intestine. This may suggest that specific approaches to restore the region-specific functions of the ileum should be considered as an effort toward fully effective treatment for patients with massive ICR.

In this study, we have described a mouse model of massive ICR, which has the potential to become a unique system to study the pathophysiology and therapy of human SBS with massive ICR. We also demonstrate by using this model that ‘functional’ adaptation mediated by phenotypical changes in the remnant intestine is not a general feature for intestinal absorption systems, as exemplified by the lack of adaptive response for vitamin B12 absorption. This may raise a significance of therapeutic strategy to compensate for the loss of region-specific functions of the resected intestine in developing a novel therapy for massive ICR, in addition to the various strategies to increase overall intestinal surface area.

## MATERIALS AND METHODS

### Mice

Male C57BL/6J mice were obtained from CLEA Japan Inc. (Tokyo, Japan) at 8-9 weeks of age. All animal experiments were performed with the approval of the Institutional Animal Care and Use Committee of Tokyo Medical and Dental University (Protocol 0170148C). We designed all experiments to minimize the number of mice used in line with principles of refinement, reduction and replacement in animal experiments, and conducted them in accordance with recognized international standards, including the principles of the Declaration of Helsinki.

### Diets and operation

Mice were kept with free access to standard diet and water before the experiment. At 12 weeks of age, the mice were randomly assigned to undergo either ICR (*n*=3-6/group) or sham operation (transection and anastomosis only, *n*=3-6/group). They were fasted for 12 h prior to surgery, but had free access to water.

Mice were anesthetized by inhalation with 2% isoflurane. A midline abdominal incision was made, and the small and large intestines were exposed in the intra-abdominal cavity. In mice undergoing ICR, the intestine was resected at the site 25 cm proximal to the ileocecal junction and the site immediately distal to the cecum in the colon. The mesentery was ligated and the intervening bowel was removed, which resulted in the excision of 75% of distal small intestine and the cecum (75% ICR). Intestinal continuity was restored with an end-to-end, single-layered anastomosis with 8-0 braid sutures. Sham operations were performed by transection and reanastomosis of the bowel at 25 cm proximal to the ileocecal junction. The abdomen was closed with 4-0 braid sutures. At the end of surgery, mice were resuscitated by subcutaneous injection of a 2 ml of 0.9% saline solution to prevent post-operative dehydration. They also received 25 mg/kg of imipenem (Sigma-Aldrich, Tokyo, Japan) subcutaneously. Mice were maintained only with water for the first 24 h post-surgery. Then they were fed with DietGel Recovery (ClearH2O, ME, USA), a nutrient fortified water gel, until they were switched back to standard diet at 8 POD.

### Study design

Survival analysis was performed for two cohorts of mice: mice that underwent 75% ICR (*n*=6) or sham operation (*n*=6). Mice were excluded if they died during surgery or within 48 h post-surgery. As the Kaplan–Meier method and log-rank test showed statistically significant difference between the two cohorts (see Fig. 1B), we did not repeat the same survival analysis. Instead, by using the body weight data on 14 POD in this analysis (*n*=6 for sham and *n*=4 for ICR) as a pilot data, we performed a power analysis retrospectively by using G*Power 3.1.9.2. (Heinrich-Heine-Universitat Dusseldorf, Germany). With the given effect size value of 5.41 and an alpha value of 0.05, a power value of 1-beta >0.999 was obtained, which indicated the sample size of 4 to 6 to be large enough. Thus, we repeated mouse experiments on two more separate occasions (independent experiments), randomly assigning more than six mice into sham group or 75% ICR group. All mice were weighed and monitored daily for survival and clinical signs. Mice with severe weight loss (≥30% of body weight before operation), hunched posture, ruffled fur, or bleeding from any orifice were sacrificed by cervical dislocation, and these events were considered lethal endpoints.

For tissue and blood sample analyses, mice were harvested at 14 POD. Blood samples were obtained by cardiac puncture during anesthesia with isoflurane (*n*=6 for each of the 75% ICR, and sham and un-operated groups), and outsourced to Oriental Yeast Co., Ltd. (Tokyo, Japan) for analysis of serum vitamin B12 levels. Tissues were harvested immediately after mice were sacrificed by cervical dislocation. Images of the removed intestines were obtained with an EOS Kiss X7 digital camera (Canon, Tokyo, Japan) equipped with a macro lens EFS (60 mm). Then, the entire lengths of intestines were measured on those digital images. As depicted in Fig. 1A, we designated in this study the first and second halves of the jejunum (J), ileum (I) and colon (C) as J1, J2, I1, I2, C1, and C2 (Fig. 1A). The remnant intestine in 75% ICR mice thus consisted of the entire duodenum (designated as D), the remaining portion of J1, and entire C1 and C2. We used the middle 1 cm of these four portions of the remnant intestine in the 75% ICR mice and the corresponding intestinal segments of sham operation mice for the following mRNA analyses. We also harvested the portion immediately proximal to each intestinal segment used for mRNA analysis for histological assays. RNA isolation and analysis were also performed with all seven segments (from D to C2 segments) of un-operated control mice. Then number of biological replicates in each analysis is indicated in the following sections.

### Histology

Intestinal tissues were fixed overnight at 4°C in 4% paraformaldehyde, sequentially dehydrated in 10, 15 and 20% sucrose in PBS, and embedded in Tissue-Tek O.C.T. compound (Sakura Finetek Japan, Tokyo, Japan). Frozen sections were stained with hematoxylin and eosin (H&E) or subjected to immunofluorescent staining by using antibodies specific for Ki67 (M7248, Dako Agilent Technologies Japan Ltd. Tokyo, Japan; used at 1:50 dilution), SLC10A2 (sc-27494, Santa Cruz Biotechnology Inc. TX, USA; 1:50 dilution), or CUBN (sc-20609, Santa Cruz Biotechnology Inc.; 1:500 dilution). In all immunofluorescent experiments, nuclei were counterstained with DAPI. For quantification of the caliber, five different perpendicular H&E sections were randomly chosen from a series of sections originating from each segment of a mouse (*n*=3 or 5 for each of the 75% ICR, sham and un-operated group per experiment), and the values were averaged and presented. We used the entire circumference of the intestinal submucosa as a measure of intestinal caliber as previously described (Dekaney et al., 2007). Intestinal calibers were measured by using ImageJ (NIH Image, MD, USA). Villus height and crypt depth were analyzed by randomly choosing well-oriented, full-length crypt-villus units (D and J1) or crypt units (C1 and C2) on H&E sections. For comparison, the values of 60 or 100 villi (D and J1) and crypts (D, J1, C1, and C2) were obtained in total by examining 20 different structural units in sections originating from individual mice (*n*=3 or 5 for each of the 75% ICR, sham and un-operated group per experiment). Quantification of Ki67+ cells on fluorescently labeled sections was conducted in the same way as for crypt depth analysis by obtaining 60 or 100 values from individual mice (*n*=3 or 5 for each of 75% ICR, sham and un-operated group per experiment). All images were acquired by using a microscope system BZ-X710 with 2× or 20× objectives (KEYENCE, Osaka, Japan). If necessary, image processing was carried out using Photoshop CS4 software (Adobe, CA, USA). Each quantitative analysis was repeated two times and thus the number of total biological replicates is eight. Statistical significance was determined by the Mann–Whitney U test (*P*<0.05).

### PCR

To investigate expression patterns of genes along the intestinal tract, intestines obtained from un-operated control mice (*n*=3) were divided into 7 segments (D, J1, J2, I1, I2, C1 and C2), and then total RNA was extracted from the middle 1 cm of each segment. Aliquots of 300 ng of total RNA were used for cDNA synthesis in 20 μl of the reaction volume. Two microliters of cDNA was assessed by qPCR using the Quantitect SYBR Green Kit (Qiagen, Tokyo, Japan) on a StepOnePlus thermocycler (Thermo Fisher Scientific K.K. Kanagawa, Japan). For comparison of gene expression in 75% ICR mice or sham operation mice, total RNA was isolated from intestinal segments (D, J1, C1, and C2) of 75% ICR mice or sham operation mice on 14 POD (*n*=6 for each) and assayed by qPCR as described above. For this comparative analysis, we also assessed expression levels of genes in the distal ileum (I2) of 12-week-old mice (*n*=6) simultaneously for reference. For all experiments, changes in gene expression were determined using the conventional double-Δ Ct method. In order to quantify expression levels of genes relative to the epithelial component rather than all intestinal tissues, we used *villin* as the reference gene of the double-Δ Ct analysis (Wittkopf et al., 2015). Primer sequences for target genes are listed in Table S1. Statistical significance was determined by the Mann–Whitney U test (*P*<0.05).

### Statistical analyses

All data were expressed as the means±s.e.m. The biological repeats are indicated by ‘*n*’. Because of the small sample size and the non-normal distribution of data, we used non-parametric Mann–Whitney U tests for statistical analyses: *P<0.05, **P<0.01 and ***P<0.001. We analyzed the survival curves using the Kaplan–Meier method and following log-rank test. All statistical analyses were carried out using GraphPad Prism version 5.0a (GraphPad Software, CA, USA).
